# Anti-*Saccharomyces cerevisiae* antibodies (ASCA) are associated with body fat mass and systemic inflammation, but not with dietary yeast consumption: a cross-sectional study

**DOI:** 10.1186/s40608-017-0164-2

**Published:** 2017-07-17

**Authors:** Anne Stine Kvehaugen, Martin Aasbrenn, Per G. Farup

**Affiliations:** 1 Department of Surgery, Innlandet Hospital Trust, Kyrre Greppsgate 11, 2819 Gjøvik, Norway; 2 Unit for Applied Clinical Research, Department of Cancer Research and Molecular Medicine, Faculty of Medicine and Health Sciences, Norwegian University of Science and Technology, Trondheim, Norway; 3 Department of Research, Innlandet Hospital Trust, Brumunddal, Norway

**Keywords:** Obesity, Anti-*Saccharomyces cerevisiae* antibodies, *Saccharomyces cerevisiae*, Baker’s yeast, Inflammation, Diet

## Abstract

**Background:**

Baker’s/brewer’s yeast, *Saccharomyces cerevisiae*, has been used as an alternative to antibiotic growth promoters to improve growth performance in animals. In humans, *Saccharomyces cerevisiae* is among the most commonly detected fungi in fecal samples and likely originates from food. Recently, an association between anti-*Saccharomyces cerevisiae* antibodies (ASCA) and obesity in humans was suggested, but the cause of the elevated ASCA levels is not clear. Our aim was to study ASCA in morbidly obese subjects and explore potential associations with anthropometrics, diet, co-morbidities and biomarkers of inflammation and gut permeability.

**Methods:**

Subjects with morbid obesity referred to a specialized hospital unit were included. Diet and clinical data were recorded with self-administered questionnaires. Main dietary sources of baker’s/brewer’s yeast (e.g. bread and beer) were used as a proxy for the intake of yeast. Laboratory analyses included ASCA, serum zonulin (reflecting gut permeability), C-reactive protein and a routine haematological and biochemical screening.

**Results:**

One-hundred-and-forty subjects; 109 (78%) female, 98 with dietary records, mean age 43 years and BMI 42 kg/m^2^ were included. The number of ASCA positive subjects was 31 (22%) for IgG, 4 (2.9%) for IgA and 3 (2.1%) for IgM. Age, body fat mass and C-reactive protein were significantly higher in IgG-positive compared to IgG-negative subjects (*P* < 0.05). A borderline significant association was found between elevated zonulin and ASCA IgG-positivity (*P* = 0.06). No association was found between yeast-containing food and ASCA IgG-positivity, or between yeast-containing food and fat mass.

**Conclusions:**

The findings indicate that ASCA IgG-positivity may be linked to the generalized inflammation commonly seen with increased adiposity, but not to dietary yeast intake. Other potential causes for the raised ASCA IgG concentrations, such as genetic predisposition, deviations in the gut microbiota and cross-reactivity of ASCA with other antigens, were not explored.

## Background

In animal husbandry, cultures of *Saccharomyces cerevisiae* (also known as baker’s and brewer’s yeast) and their cell wall components have appeared as promising alternatives to Antibiotic Growth Promoters in order to improve growth performance, feed intake and feed efficiency [1, 2]. Speculatively, similar effects could be present in humans. In a study from 2015, Salamati et al. investigated associations between anti-*Saccharomyces cerevisiae* antibodies (ASCA) IgG and IgA and obesity [1]. In this study, ASCA levels were higher in morbidly obese than in normal weight subjects, and there was a correlation between ASCA and BMI [1]. The cause of the elevated ASCA levels is however not clear.

*Saccharomyces cerevisiae* is among the most commonly detected fungi in human fecal samples and it presumably originates from food [3]. In human diet, bread is one of the main dietary sources of this yeast (baker’s yeast), along with beer (brewer’s yeast). The scientific literature does however not support a relationship between bread consumption and obesity, although consumption of white bread, as opposed to whole grain bread, might be associated with excess abdominal fat and impaired weight loss [4]. To our knowledge, an association between ASCA and yeast-containing food in relation to obesity remains to be explored.

*Saccharomyces cerevisiae* also has the ability to grow at body temperature and to respond to the gut environment, suggesting that it may play a role in gut microbial ecology [3]. A growing body of evidence suggests that deviations in the gut microbiota and increased intestinal permeability are associated with obesity and the metabolic inflammation that may accompany excess adiposity [5]. A recent recognition is that specific anti-microbial antibodies may as well be raised in obesity and associated co-morbidities, such as diabetes and glucose intolerance [6]. Increased gut permeability, with the potential translocation of *Saccharomyces cerevisiae* or related luminal antigens, could thus be another cause of elevated ASCA concentrations in obesity.

The aims of the present paper were to study ASCA in subjects with morbid obesity in a larger study than the previously published study by Salamati et al. [1], and to study associations between ASCA and dietary intake, obesity-related co-morbidities and biomarkers of inflammation and gut permeability.

## Methods

The present study used data from a cross-sectional study at Innlandet Hospital Trust, Gjøvik, Norway. Patients referred for morbid obesity (BMI ≥ 40 kg/m^2^ or BMI ≥ 35 kg/m^2^ with obesity related complications (e.g. diabetes, hypertension, sleep apnoea, musculoskeletal problems) from December 2012 to September 2014 were asked to participate in the study. The patients were evaluated for bariatric surgery or non-surgical treatment of obesity. The patients had not started the weight reducing intervention at the time of study inclusion. The study used a large range of questionnaire data collected at the time of inclusion, with a case report form and a food frequency questionnaire (FFQ). Supplementary information was collected from the patient records. For the present sub-study, patients with organic gastrointestinal disorder, former major gastrointestinal surgery, unsatisfactory filled in case-report form or lacking blood samples for the biobank were excluded.

### Case report form

Included age, gender, height, weight, education, marital status, occupation, employment, physical activity, smoking, alcohol consumption, perception of overall state of health, musculoskeletal complaints, mental distress (Hopkins Symptom Checklist 10 (HSCL-10): a score above 1.85 indicates mental distress (range: 1–4)) [7] and other co-morbidities. All these questions have been validated and used in accordance with The Norwegian Institute of Public Health’s population based surveys. Additionally, Irritable bowel syndrome (classified according to the Rome III criteria) [8], perceived food intolerance in association with gastrointestinal symptoms and the following health scores were also included in the case-report form: well-being (WHO-5; score: 0–100) [9], sense of humour (SHQ-6; score: 6–24) [10], fatigue (score: 1–7) [11], sleepiness (Epworth; score: 0–24) [12] and self-esteem (Rosenberg; score: 10–40) [13]. In case of missing data within a scoring algorithm, the data were treated as follows: If a patient answered less than 50% of the questions, the health score was evaluated as blank/missing (Rosenberg: 1 patient. Humor: 1 patient), otherwise the missing values (0%-0.57% for each tool) were assigned the least pathological option among the alternatives.

### Diet

The patients were asked to recall their habitual diet during the last year by answering a FFQ, prepared and validated by the University of Oslo [14, 15]. Daily intake of food, nutrients and energy was calculated by Department of Nutrition at the University of Oslo by their in-house calculation program (KBS, version 7.3, food database AE-14). The food composition database in the calculation program is based on the official Norwegian food composition table from 2016 [16] and is supplemented with additional food items. Intakes from dietary supplements were included in the calculations. Bread, buns and other baked goods that typically contain (baker’s) yeast, and beer (brewer’s yeast), were used as a proxy for the intake of yeast. Calculation of total yeast intake was performed by summarizing the intakes of these food items for each patient.

### Laboratory analyses

The patients were asked to fast the night before the blood draw. Serum and EDTA plasma were then collected and immediately analysed or stored at −80 °C for later analysis. ASCA; IgG, IgA and IgM against *Saccharomyces cerevisiae* mannan, were determined with an Enzymatic Linked Immunosorbant Assay according to the manufacturer’s instructions (IgG and IgA: DRG International, Inc., and IgM: BIOGEMA Košice). A positive test result was defined as ≥10 U/mL for IgG and IgA and ≥33 U/mL for IgM, as suggested by the manufacturers. The ASCA analyses were performed by Vitas AS, Oslo, Norway. Serum zonulin, a marker of intestinal permeability (reference range < 48 ng/mL) was measured by Lab 1, Sandvika, Norway (SYNLAB group; SYNLAB International GmbH) using an Enzymatic Immunosorbant Assay. Serum C-reactive protein (CRP) was analysed as part of a routine haematological and biochemical screening, including a total number of 44 parameters (e.g. red blood cell indices, haemoglobin, iron status parameters, leukocytes, thrombocytes, markers of kidney and liver function, thyroid hormones, blood lipids, glucose, HBA1C, electrolytes and vitamins).

### Statistical analyses

Data analyses were performed with IBM SPSS Statistics for Windows, Version 23 (IBM Corp., Armonk, N.Y., USA). Associations between variables were tested with logistic and linear regression models. Adjustments were made for age, gender and BMI, as these were considered clinically relevant co-variates. If another anthropometrical variable was included in the model (i.e. height, weight, fat mass or fat percentage) adjustment for BMI was not performed. Ordinal independent variables without pre-defined categories (e.g. health indexes with a lower and upper total score) were treated as continuous variables in the model. Interactions were tested by adding an interaction term to the regression model. Significance level was set at a *P*-value <0.05. The 95% confidence intervals (CI) for the prevalence of positive ASCA IgG, IgA and IgM levels were obtained using StatXact 10 (Cytel Software Corporation, Cambridge MA, USA).

### Post-hoc tests

Due to initial findings from association analyses between ASCA and anthropometrical data, we included an estimate of body fat percentage using the formula developed by Gallagher et al. [17], and estimated body fat mass in kg (estimated body fat percentage [17] * weight in kg/100).

## Results

**Fig. 1:**
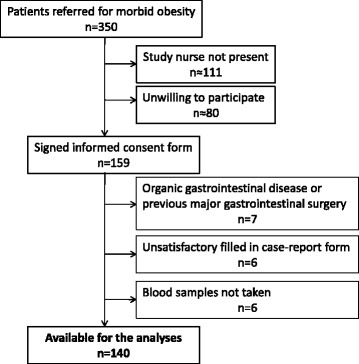
Inclusion diagram

Figure 1 shows the number of included and excluded patients. Of 159 included patients, 140 patients (male/female 31/109, mean age 43.1 years) were available for the analyses. Of these, 98 had completed the FFQ. Table 1 gives the patients’ characteristics.

**Table 1:** Demographical and clinical data for the study population ^a^

	All patients, *n* = 140 ^b^
Age, years	43.1 (8.7)
Gender (male/female)	31 (22%)/109 (78%)
Ethnic Nordic background	138 (99%)
Height, cm	169 (149–197)
Weight, kg	120 (90–192)
BMI, kg/m^2^	41.6 (35–55)
Body fat, percentage of body weight	46.8 (31–51)
Body fat mass, kg	54.1 (39–81)
Smoking (never/former/current)	55 (39%)/60 (43%)/25 (18%)
Previous or present co-morbidity	
Diabetes mellitus	26 (19%)
Polycystic ovary syndrome	7 (6.4%)
Hypertension	46 (33%)
Angina pectoris	1 (0.7%)
Myocardial infarction	2 (1.4%)
Stroke	3 (2.1%)
Fibromyalgia	25 (18%)
Hypothyroidism	18 (13%)
Hyperthyroidism	1 (0.7%)
Gallstones	21 (15%)
Irritable bowel syndrome (Rome III)	35 (26%)
Chronic obstructive pulmonary disease	3 (2.1%)
Asthma	34 (24%)
Hay fever	19 (14%)
Osteoporosis	0 (0%)
Asked for help for psychological problems	28 (20%)
Mental distress (HSCL-10 score > 1.85)	34 (24%)

^a^Data are expressed as mean (SD), median (min-max) and n (%)

^b^All data are based on *n* = 140 with the following exceptions: 1) Irritable bowel syndrome: *n* = 135. 2) Polycystic ovary syndrome; *n* = 109 (females only)

Thirty-one patients (22%; 95% CI: 16 to 30%), 4 patients (2.9%; 95% CI: 0.8 to 7.1%) and 3 patients (2.1%; 95% CI: 0.4 to 6.1%) had ASCA IgG, IgA and IgM levels above the respective reference values. Because of the high frequency of IgG-positivity, further analyses were restricted to comparisons between patients with ASCA IgG above and below 10 U/mL. The results from these analyses showed significant and direct associations between ASCA IgG-positivity and age, height, weight, body fat mass and serum CRP respectively (Tables 2 and 3). Elevated zonulin concentrations (≥ 48 ng/ml) were more frequently detected in the IgG-positive compared to the IgG-negative group, but the difference did not reach statistical significance (Table 3). Moreover, using linear regression, significant positive associations were found between body fat mass (independent variable) and CRP; B = 0.14 (0.01–0.28), *P* = 0.04, and between zonulin dichotomized at 48 ng/ml (independent variable) and CRP; B = 3.28 (1.10–5.46), *P* < 0.01, but not between zonulin dichotomized (independent variable) and body fat mass; B = 0.07 (−2.76–2.91), *P* = 0.96. No associations were found between ASCA IgG-positivity and the dietary intake data, neither with respect to food groups, nor nutrients. In particular, no associations were found between ASCA IgG and yeast-containing food, energy-yielding nutrients or total energy intake (Table 2). Additionally, no associations were found between the total intake of yeast-containing food (independent variable) and the obesity indices BMI (B = 0.003, *P* = 0.38), body fat percentage (B = 0.001, *P* = 0.60) or body fat mass (B = 0.006, *P* = 0.41). Also, no interactions were found between ASCA IgG (above and below 10 U/ml) and the total intake of yeast-containing food with respect to these anthropometrical outcomes (all *P*-values >0.05). The two groups (ASCA IgG-positive versus ASCA IgG-negative) were otherwise similar in terms of physical activity, smoking, alcohol consumption, co-morbidities and other clinical and biochemical characteristics, with the following exceptions: HSCL-10 score > 1.85 (indicating mental distress) was significantly more prevalent (Table 2), and serum albumin and serum protein were significantly higher (Table 3) among the IgG-positive compared to the IgG-negative subjects. Blood mean corpuscular volume and mean corpuscular hemoglobin were significantly lower among the IgG-positive compared to the IgG-negative subjects, whereas no significant differences were found in related parameters (e.g. hemoglobin and ferritin) between the two groups (Table 3). The variables assumed to be of main clinical interest and all statistically significant variables are displayed in Tables 2 and 3.

**Table 2:** Clinical and dietary data; comparing ASCA IgG ≥ 10 U/mL to IgG < 10 U/mL ^a^

	IgG ≥ 10 U/mL	n	IgG < 10 U/mL	n	Adjusted for age, gender and BMI ^b^	*P-value*
					Odds Ratio (95% CI)	
Age, years	45.3 (7.9)	31	42.5 (8.8)	109	1.06 (1.00–1.11)	*0.04* *
Gender (male/female)	9 (29%)/22 (71%)	31	22 (20%)/87 (80%)	109	1.29 (0.49–3.36)	0.61
Height, cm	172 (161–197)	31	168 (149–192)	109	1.09 (1.01–1.16)	*0.02* ^c,^ *
Weight, kg	128 (95–183)	31	118 (90–192)	109	1.04 (1.01–1.07)	*<0.01* ^c,^ *
BMI, kg/m2	42.8 (3.9)	31	41.9 (3.8)	109	1.10 (0.98–1.24)	0.10
Body fat, percentage of body weight	48 (33–50)	31	47 (31–51)	109	1.22 (0.94–1.58)	0.14 ^c^
Body fat mass, kg	59 (43–75)	31	53 (39–81)	109	1.07 (1.02–1.13)	*0.01* ^c,^ *
Dietary intake						
Bread, g	148 (24–267)	23	174 (48–444)	75	1.00 (0.99–1.00)	0.39
Beer, g	0 (0–180)	23	0 (0–140)	75	1.01 (0.99–1.02)	0.35
Bread, buns and other baked goods + beer, g	172 (24–387)	23	187 (53–1060)	75	1.00 (0.99–1.00)	0.39
Energy, kJ	9293 (5100–20,111)	23	9737 (4830–21,816)	75	1.00 (1.00–1.00)	0.58
Carbohydrates, g	251 (79–475)	23	247 (94–903)	75	1.00 (0.99–1.00)	0.60
Proteins, g	109 (64–177)	23	108 (43–213)	75	1.00 (0.99–1.02)	0.99
Fats, g	82 (27–231)	23	93 (38–283)	75	1.00 (0.99–1.01)	0.61
Alcohol, g	0.6 (0–10)	23	1.3 (0–21)	75	0.93 (0.79–1.08)	0.33
Physical activity (light) ^d^		31		109		
< 1 h/week (reference)	7 (23%)		30 (28%)			
1–2 h/week	13 (42%)		43 (39%)		1.54 (0.53–4.54)	0.43
≥ 3 h/week	11 (35%)		36 (33%)		1.69 (0.55–5.22)	0.36
Physical activity (strenuous) ^d^		31		109		
< 1 h/week (reference)	17 (55%)		66 (61%)			
≥ 1 h/week	14 (45%)		43 (39%)		1.23 (0.54–2.80)	0.62
Smoking		31		109		
Never (reference)	13 (42%)		42 (39%)			
Former	14 (45%)		46 (42%)		0.90 (0.36–2.24)	0.83
Current	4 (13%)		21 (19%)		0.78 (0.21–2.83)	0.70
Previous or present co-morbidity						
Hypertension	10 (32%)	31	36 (33%)	109	0.65 (0.25–1.70)	0.38
Diabetes mellitus	9 (29%)	31	17 (16%)	109	1.98 (0.74–5.32)	0.18
Hypothyroidism	6 (19%)	31	12 (11%)	109	1.89 (0.62–5.74)	0.26
Irritable Bowel Syndrome	8 (27%)	30	27 (26%)	105	1.25 (0.47–3.28)	0.66
Mental distress (HSCL-10 score > 1.85)	12 (39%)	31	22 (20%)	109	2.53 (1.04–6.17)	*0.04* *

^a^Data are expressed as mean (SD), median (min-max) and n (%)

^b^Based on logistic regression with ASCA IgG dichotomized at cut-off 10 as the dependent variable and age, gender, BMI and one by one of the column variables as independent variables

^c^Adjusted for age and gender only

^d^Categories are collapsed as compared to originally four categories; no significant differences between the groups were seen when all original categories were included (data not shown)

*Statistically significant *P* < 0.05

**Table 3:** Biochemical data; comparing ASCA IgG ≥ 10 U/mL to IgG < 10 U/mL ^a^

	IgG ≥ 10 U/mL	n	IgG < 10 U/mL	n	Adjusted for age, gender and BMI ^b^	*P-value*
					Odds Ratio (95% CI)	
S-CRP, mg/L	7.0 (0–43.0)	31	5.0 (1.0–28.0)	109	1.07 (1.00–1.14)	*0.04* *
S-Zonulin, ng/ml	64.1 (17.4–117)	31	52.4 (20.1–186)	105	1.00 (0.99–1.01)	0.89
S-Zonulin ≥48 ng/ml	24 (77%)	31	66 (63%)	105	2.51 (0.95–6.66)	0.06
S-Glucose, mmol/L	5.9 (4.6–23.2)	31	5.6 (4.0–21.5)	109	1.06 (0.94–1.20)	0.31
S-HBA1C, %	5.6 (4.7–11.5)	31	5.4 (4.5–11.1)	109	1.26 (0.96–1.67)	0.10
S-Cholesterol, mmol/L	5.1 (0.9)	31	5.1 (1.0)	109	0.99 (0.64–1.54)	0.97
S-HDL, mmol/L	1.1 (0.7–2.0)	31	1.1 (0.4–2.2)	109	0.53 (0.13–2.18)	0.38
S-LDL, mmol/L	3.3 (0.9)	31	3.4 (0.8)	109	0.97 (0.60–1.57)	0.90
S-Albumin g/L	45.0 (39.0–52.0)	31	44.0 (39.0–52.0)	109	1.20 (1.02–1.42)	*0.03* *
S-Total protein, g/L	72.9 (3.2)	31	71.2 (3.8)	109	1.13 (1.01–1.28)	*0.04* *
B-MCV, fL	87.3 (4.8)	31	89.2 (4.2)	109	0.87 (0.78–0.96)	*<0.01* *
B-MCH, pg	29.0 (26.0–34.0)	31	31.0 (25.0–35.0)	109	0.72 (0.56–0.92)	*0.01* *
B-Hemoglobin, g/dL	14.6 (1.2)	31	14.4 (1.0)	109	1.23 (0.77–1.96)	0.39
S-Ferritin, μg/L	94.0 (17.0–466)	31	99.0 (7.0–584)	109	1.00 (0.99–1.00)	0.78

^a^Data are expressed as mean (SD), median (min-max) and n (%)

^b^Based on logistic regression with ASCA IgG dichotomized at cut-off 10 as the dependent variable and age, gender, BMI and one by one of the column variables as independent variables

*Statistically significant *P* < 0.05

## Discussion

In the present study, including 140 patients, 22% had a positive ASCA IgG test result. This frequency of ASCA positivity in subjects with morbid obesity was at least of the same order of magnitude as the previous publication by Salamati et al. [1]. Our study did however not show an association between ASCA and BMI. This might have been due to the fact that our study included subjects with morbid obesity only, limiting interpretations of results to this BMI range. On the other hand, an association was detected between ASCA IgG-positivity and body fat mass, suggesting that in a population of subjects with morbid obesity, total amount of body fat is a better predictor of ASCA than the relative amount of body fat.

The findings of a relationship between ASCA and adiposity in humans are interesting considering the use of *Saccharomyces cerevisiae* as a growth promoter in the animal husbandry [1, 2]. Consumption of the main dietary sources of baker’s/brewer’s yeast was however not reflected by the plasma levels of ASCA IgG in our study, indicating that ASCA IgG formation was unrelated to yeast intake. Additionally, we did not find any association between the total intake of these yeast-containing foods and the anthropometrical indices BMI, body fat mass or body fat percentage. This finding was independent of ASCA status, suggesting that effect modification was unlikely. In all, these results do not support a causal relationship between the consumption of baker’s/brewer’s yeast and obesity in humans.

The amount and type of yeast in the gut may also depend on the nutrient availability within the gut. A study which reported associations between nutrients and the presence of fungal populations in fecal samples did however not find any relationship between *Saccharomyces* and the nutrient composition of the diet, either recent or long term diet [18]. In the present study, there were no associations between ASCA IgG and either energy yielding nutrients or total energy intake.

In addition to the association between ASCA IgG and body fat mass, our study showed an association between ASCA IgG positivity and serum CRP. Obesity is associated with a state of low-grade inflammation, including increments in serum CRP [19]. There is also some evidence suggesting a positive association between obesity and immunoglobulin levels [20–22], including a study which reported concomitant elevations of anti-food IgG and CRP in obese compared to normal weight juveniles [22]. This relates to our finding that ASCA IgG-positivity, CRP and body fat mass were all inter-correlated. Together with a study which showed that IgG directed against specific enteric bacterial antigens may be related to obesity and the associated metabolic inflammation [6], these results indicate that certain dietary or other luminal (microbial) antigens may be linked to the generalized inflammation commonly seen with increased adiposity. In contrast to our finding, Salamati et al. [1] did not demonstrate a correlation between serum CRP and ASCA values. The higher sample size in our study and some differences in inclusion criteria may have accounted for this difference. A limitation is that neither we, nor the study by Salamati et al. [1], measured other inflammation markers in addition to CRP. Adipose tissue has many endocrine functions and a large range of signaling molecules may be released either from the adipose tissue and/or immune cells within the adipose tissue, implying that other inflammation markers could be the ones that are mechanistically linked to ASCA IgG.

Evidence suggests an association between increased gut permeability and systemic inflammation in obesity, and the association may be bi-directional [5]. Moreover, high concentrations of ASCA have been found in Crohn’s disease [23, 24] and other diseases associated with increased gut permeability, such as celiac disease and type 1 diabetes mellitus [25–27]. A “leaky gut”, with increased translocation of *Saccharomyces cerevisiae* (or related antigens) could thus be one potential explanation for the increased ASCA IgG concentrations and their association with CRP. In the present study we measured zonulin, a marker of tight junction permeability in the gut [26]. We found that elevated zonulin levels were more frequent in subjects with ASCA IgG-positivity than in subjects negative for ASCA IgG and the elevated levels correlated significantly with increasing concentrations of CRP. However, the observed difference in zonulin between the ASCA IgG-positive and the ASCA IgG-negative group did not reach statistical significance. A study of patients with Crohn’s disease also showed a non-significant trend between elevated ASCA IgG and intestinal permeability, but similar to our study, could not demonstrate a clear cut association [23]. Thus it appears that the elevated ASCA concentrations cannot be explained solely by an increased antigenic challenge due to a leaky gut. Genetic pre-disposition, deviations in the gut microbiota and cross-reactivity of ASCA with other antigens could be other causes for the raised ASCA IgG concentrations and their association with body fat mass and/or inflammation in a sub-group of obese subjects. These aspects were not explored in the present study, but could be directions for future research.

### Strenghts and limitations

Strength of the study is that it was based on a well characterized population with the assessment of a wide range of demographic, clinical and biochemical data, thereby reducing the risk of unknown confounders. A limitation is that the many variables that have been addressed also increased the risk of a type I statistical error. Information about the use of medications was however incomplete. Thus, we cannot rule out that the use of certain drugs could have influenced our findings. Information regarding certain autoimmune conditions was also lacking. In the present study, the 95% CI for the proportion of IgG positive samples ranged between 16% and 30%, which is comparable to the frequency of IgG positivity found in diseases of autoimmune origin, such as type 1 diabetes mellitus [27], systemic lupus erythematosus [28] and microscopic colitis [29], but lower than in Crohn’s disease [30]. No significant association was however detected between ASCA and disorders such as hypothyroidism or diabetes (the majority of the diabetic patients had type II diabetes). Moreover, patients with organic gastrointestinal disorders or former major gastrointestinal surgery were excluded from the study.

The lack of a normal weight control group represents a limitation with respect to reference values for ASCA, but based on the aforementioned studies [27–30], the frequency of IgG positivity in our study was higher than what would be expected in healthy control subjects, in whom the reported prevalence ranged between 0 and 5.0% [27–30]. This is also in accordance with the previous publication of ASCA and obesity by Salamati et al., in which none of the normal weight controls were ASCA positive [1].

Other methodological considerations of the present study include the dietary assessment tool and our definition of yeast intake: Firstly, calculation of the exact intake of this yeast was not possible based on our FFQ. We therefore chose to focus on two major dietary sources of *Saccharomyces cerevisiae*, namely bread, buns and other baked goods (baker’s yeast) and beer (brewer’s yeast). Results from our study did however not reveal any significant associations between ASCA and other food groups or between ASCA and total alcohol consumption. Secondly, because the FFQ that we applied was designed to study the habitual diet during the last year, conclusions cannot be made regarding the impact of more recent diet or diet early in life. Also, recalling food intake over a year may be inaccurate, but with respect to the main dietary sources of yeast, we believe that significant fluctuations in intake during the year were unlikely. This is because bread is regarded a staple food in Norway that is regularly consumed all year round. Beer on the other hand probably contributes less to the total intake of yeast as compared to bread.

## Conclusions

The findings indicate that ASCA IgG-positivity, which was detected in a substantial proportion of obese subjects and was associated with body fat mass, may be linked to the generalized inflammation commonly seen with increased adiposity. The study did not show an association between the habitual intake of food containing baker’s/brewer’s yeast and either body fat mass or ASCA, weakening the hypothesis that dietary yeast is causally related to obesity. The impact of more recent diet or diet early in life, genetic susceptibility, gut microbial composition and cross-reactivity of ASCA with other antigens were not addressed in the present study and could be directions for future research. The finding of a borderline association between ASCA IgG and intestinal permeability also needs further elucidation.

## Abbreviations

ASCA: Anti-*Saccharomyces cerevisiae* antibodies
CRP: C-reactive protein
FFQ: Food Frequency Questionnaire
HSCL-10: Hopkins Symptom Checklist 10
